# Beyond the Known: Expanding the Clinical and Genetic Spectrum of Rare RPL13-Related Spondyloepimetaphyseal Dysplasia

**DOI:** 10.3390/ijms26146982

**Published:** 2025-07-20

**Authors:** Daria Gorodilova, Elena Dadali, Vladimir Kenis, Evgenii Melchenko, Daria Akimova, Maria Bulakh, Anna Orlova, Maria Orlova, Olga Shatokhina, Evgeniya Melnik, Marc Baud’huin, Mikhail Skoblov, Sergey Kutsev, Tatiana Markova

**Affiliations:** 1 Research Centre for Medical Genetics, Moskvorechye St., 1, 115522 Moscow, Russia; 2 H. Turner National Medical Research Center for Children’s Orthopedics and Trauma Surgery, Parkovaya 64–68, Pushkin, 196603 Saint-Petersburg, Russia; kenis@mail.ru (V.K.);; 3 Nantes Université, Univ Angers, CHU Nantes, INSERM, CNRS, CRCI2NA, F-44000 Nantes, France; marc.baudhuin@univ-nantes.fr

**Keywords:** *RPL13*, skeletal dysplasia, spondyloepimetaphyseal dysplasia, SEMD, incomplete penetrance, ribosomopathy

## Abstract

Spondyloepimetaphyseal dysplasia type Isidor-Toutain (RPL13-SEMD) is an autosomal dominant skeletal dysplasia caused by heterozygous pathogenic variants in the *RPL13* gene, encoding the ribosomal protein eL13. To date, 13 pathogenic variants in *RPL13* have been reported, all clustering within intron 5 and exon 6, suggesting this hotspot region is critical for the function of ribosomes in skeletal tissues. Here, we present clinical and radiological characteristics of seven individuals, five children and two adults, from four unrelated families with RPL13-SEMD caused by two novel variants (c.477+5G>C and c.539_541del) and two previously reported variants (c.477+1G>C and c.548G>A) in *RPL13*. RNA analysis demonstrated that c.477+5G>C leads to a 54-nucleotide extension of exon 5, resulting in an 18-amino acid insertion. The phenotypic spectrum ranged from mild manifestations, such as Blount-like tibial deformity without significant short stature or Perthes-like femoral epiphyseal changes, to severe skeletal deformities with disproportionate short stature, accompanied by extraskeletal features (e.g., penoscrotal hypospadias, coccygeal abnormalities). For the first time, we describe Blount-like tibial deformity as a feature of this dysplasia, which resolves with age. Our study provides additional insights into the clinical, radiological, and genotypic features of RPL13-SEMD through detailed analysis of patients and their affected relatives.

## 1. Introduction

Spondyloepimetaphyseal dysplasia type Isidor-Toutain (OMIM#618728) is a rare autosomal dominant skeletal dysplasia caused by heterozygous variants in the *RPL13* gene. Its early clinical features manifest in infancy and are characterized by disproportionate short stature, abnormal vertebral bodies, significant changes in the metaphyses and epiphyses of the proximal femurs, and varus deformity of the lower limbs. Two of these patients were first reported by Isidor B. et al. in 2013 [1]. The disease etiology was identified by Le Caignec C. et al. in 2019 through trio exome sequencing of four unrelated individuals [2]. Their study identified heterozygous variants in *RPL13*, located on chromosome 16q24.3, in all patients. The gene encodes eL13 protein, which is a part of the 60S ribosomal large subunit. To date, the disease pathogenesis remains incompletely understood, but functional studies suggest that the protein may play a crucial role in translating particular mRNAs in chondrocytes and/or osteoblasts of the growth plate [2,3]. To date, 13 pathogenic nucleotide variants in *RPL13* have been reported, all clustering within the hotspot region comprising intron 5 and exon 6 [2,3,4,5]. This region is proposed to be essential for proper ribosomal function in skeletal tissues [3]. Therefore, based on the known etiopathogenetic mechanisms, RPL13-SEMD is classified as a ribosomopathy—a group of disorders caused by ribosomal protein dysfunction, impaired mRNA translation, and dysregulated ribosome biogenesis [4,5,6,7,8]. Ribosomopathies demonstrate significant clinical polymorphism linked to the predominant involvement of specific tissues in particular disorders [9]. Among the most well-characterized ribosomopathies are Diamond–Blackfan anemia, Shwachman–Diamond syndrome, cartilage–hair hypoplasia, and Treacher Collins syndrome, which are characterized by a combination of skeletal and extraskeletal manifestations affecting multiple organs or systems. Current evidence suggests that RPL13-SEMD appears to be the only known isolated skeletal dysplasia primarily affecting large joints and caused by ribosomal dysfunction [2,10]. As there is still no convincing explanation for the tissue-specificity phenomenon of ribosomopathies, and given the extreme rarity of this condition, describing the clinical and genetic characteristics of newly identified patients remains highly relevant. This not only expands our knowledge of the clinical manifestations of RPL13-SEMD but also provides valuable insights into the pathogenetic mechanisms underlying the disease.

The aim of this study is to analyze the clinical, genetic, and radiological characteristics of seven individuals from four unrelated families with SEMD type Isidor-Toutain caused by two novel and two previously reported variants in the *RPL13* gene.

## 2. Results

### 2.1. Molecular and Splicing Analysis

**Figure 1:**
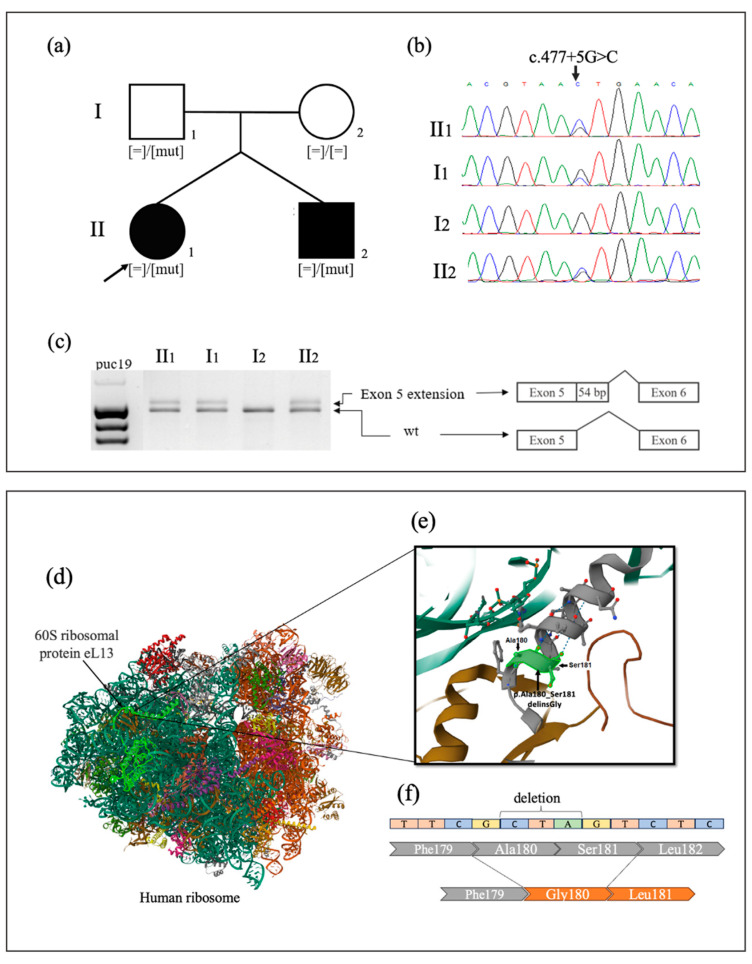
(**a**) Family pedigree analysis of P2. (**b**) Segregation analysis of c.477+5G>C variant detected in *RPL13* by Sanger sequencing. (**c**) Splicing assay of the c.477+5G>C variant identified in P2, her affected twin brother, and her presumably unaffected father. Gel electrophoresis showing the presence of an abnormal longer fragment. Sanger sequencing of the aberrant amplified products revealed an extension of exon 5 by 54 nucleotides. (**d**) General organization of the human ribosome protein (6OLG); 60S ribosomal protein eL13 is indicated in bright green. (**e**) In window above is demonstrated p.Ala180_Ser181delinsGly variant localization; amino acids Ala180 and Ser181 are a part of alpha helix H7. (**f**) Deletion of the nonpolar neutral alanine and polar serine, along with the insertion of a single nonpolar glycine residue, results in the loss of a hydrophilic amino acid and may induce conformational changes in the protein’s secondary structure.

Four heterozygous variants in the *RPL13* gene were identified in probands, including two novel variants, c.477+5G>C and c.539_541del, p.(Ala180_Ser181delinsGly), and two previously reported variants, c.477+1G>C and c.548G>A, p.(Arg183His) [4,5]. The novel splice variant c.477+5G>C was identified not only in proband 2 (P2) but also in her affected twin brother and presumably unaffected father, suggesting incomplete penetrance, as previously reported in the literature [3]. To determine the splicing effect of the novel variant c.477+5G>C located in intron 5, RT-PCR analysis was performed on total RNA extracted from blood samples of the proband, her twin brother, and her father. Sanger sequencing of the amplified products detected both the reference and an aberrant mRNA isoform containing a 54-nucleotide extension of exon 5, observed in a state of allelic imbalance. Therefore, it was confirmed that the c.477+5G>C variant leads to an 18-amino-acid insertion p.(Asn159_Val160ins18), consistent with the effects previously demonstrated for the three reported variants: c.477+1G>T, c.477+2T>C, and c.477+3A>T [2,5] (Figure 1). Consequently, this variant was classified as likely pathogenic (PM2, PM4, PM1) according to ACMG guidelines [11].

The novel de novo variant c.539_541del located in exon 6 of *RPL13* identified in proband 4 (P4) results in the in-frame deletion of two amino acids and the insertion of one—p.(Ala180_Ser181delinsGly). It was classified as pathogenic (PS2, PM1, PM2, PM4) according to ACMG guidelines [11]. This variant is located in a highly conserved region of the eL13 protein within the alpha helix (H7), which plays a critical role in ribosome assembly (Figure 1) [2,3]. Jacob et al. described a cluster of missense variants, p.17x-18x, located in the conserved region of alpha helix 7 (H7). This region, along with helix H1 and other parts of the eL13 protein, is responsible for binding to 28S rRNA. It is suggested that alterations within this cluster of missense variants may disrupt the protein’s interaction with rRNA [4].

### 2.2. Clinical and Radiographic Findings

The most common features were mild to moderate short stature, irregularities of the vertebral endplates, Perthes-like fragmentation of the femoral epiphysis, and Blount-like tibial deformity with severe lower limb varus deformity, which made differential diagnosis challenging.

#### 2.2.1. Family 1

A 7-year-old girl (proband 1; P1), the only child in the family, was referred to a geneticist due to progressive lower limb deformities and suspicion of MATN3-related multiple epiphyseal dysplasia (MATN3-MED). The child was delivered via cesarean section during the first pregnancy in complete breech position. Birth weight was 3150 g (−0.7 SD), length was 50 cm (0.06 SD), and the Apgar score was 8/9. At 3 months, hip dysplasia and an absence of the femoral head ossific nucleus were identified. Following the onset of independent walking at 14 months, varus deformity of the lower limbs developed. It progressed by the age of 3, leading to her height being at the lower limit of normal—89 cm (−2 SD). Radiographs of the knee joints revealed a symmetrical, beak-like step deformity of the tibial metaphyses. These findings suggested the possibility of a rickets-like disorder or Blount disease [12]. Laboratory tests of blood biochemical markers, such as calcium, phosphorus, alkaline phosphatase, parathyroid hormone, vitamin D, and urinary calcium and phosphorus excretion, showed no deviations from normal values. At the age of 3, correction of genu varum was performed using temporary hemiepiphysiodesis.

**Figure 2:**
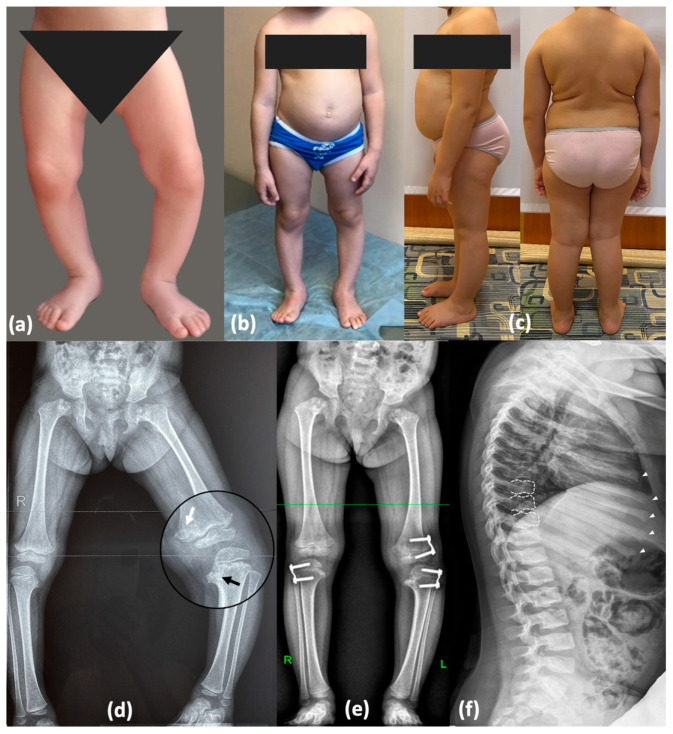
Phenotype and skeletal radiographies of P1 at various ages: Photos taken (**a**) at 1.5 years (before surgery); (**b**) at 3 years 7 months—after temporary hemiepiphysiodesis; (**c**) at 7 years 6 months—significant improvement was observed following plate removal and axial correction after 1.5 years. (**d**) Varus deformity of the lower limbs (3 y.o., before surgery)—severe on the left side and mild on the right side. Metaphyseal irregularities predominantly in the medial parts of the femur and tibia with mild “chondromatous” changes in the femur (white arrow) and Blount-like tibia vara (black arrow). (**e**) Correction of the deformities with the “guided growth” technique (3 years 7 months). Perthes-like fragmentation of the capital femoral epiphysis and widening of the femoral neck. (**f**) Irregularities of the vertebral endplates (white dotted lines) and enlarged distal parts of the ribs with rickets-like edges (white arrowheads).

At the age of 7 years, her height was 115 cm (−1.31 SD) and her weight was 35 kg (1.99 SD). Detailed examination revealed a waddling gait, cervicothoracic kyphosis, lumbar hyperlordosis, a protruding abdomen, hand joint hypermobility, hyperextended knees, valgus deformity of the right lower limb, mild varus deformity of the left lower limb, and equino-abducto-plano-valgus foot deformity. Analysis of radiographic findings revealed age-related evolution of signs caused by abnormal ossification of the hip and knee joints, which contributed to similarities with a range of disorders included in the differential diagnosis, as detailed in Figure 2 and Supplementary Figure S1.

A rare form of SEMD, caused by previously reported splice variant c.477+1G>C in *RPL13*, was diagnosed in P1 and her mother through trio whole-genome sequencing. The variant was inherited from her affected mother, who has severe osteoarthritis of the lower limbs. The clinical and radiological findings of the proband’s mother are provided in Supplementary Table S1 and Figure S2.

#### 2.2.2. Family 2

**Figure 3:**
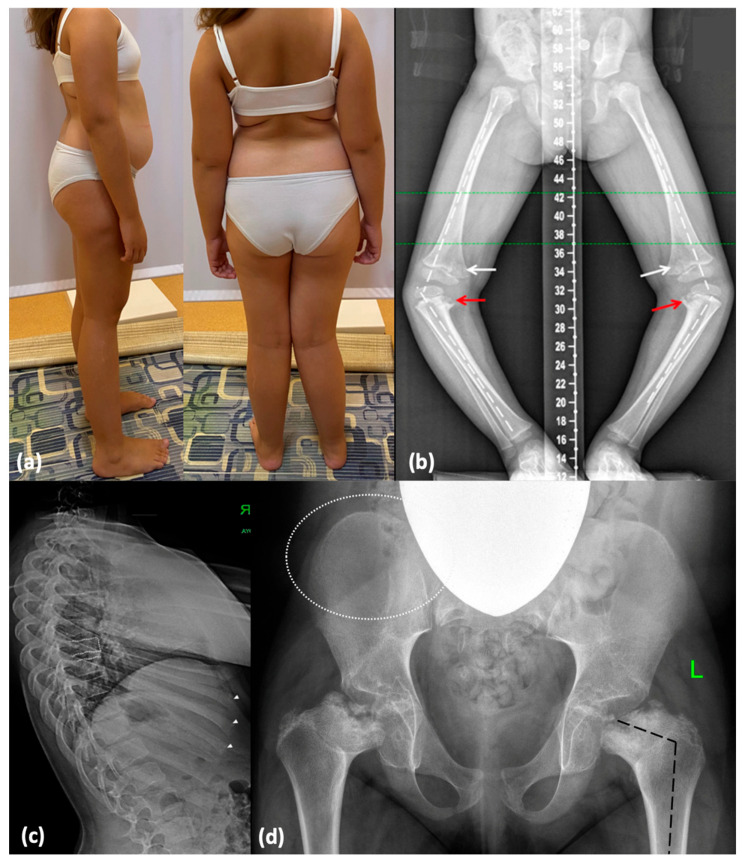
Phenotype and skeletal radiographies of P2 at various ages: (**a**) A photo taken at 9 years and 4 months. (**b**) Severe varus deformity (white broken lines) secondary to the Blount-like tibia vara (red arrows) and metaphyseal irregularities predominantly in the medial parts of the femur (white arrows) (3 y.o., before surgery). (**c**) Irregularities of the vertebral endplates are mildly represented (white dotted lines), and distal parts of the ribs are mildly enlarged (white arrowheads). (**d**) Coxa vara (black broken lines) and abnormal ossification of the femoral neck and head; minimal lace-like appearance of the iliac crests (indicated by white dotted lines).

A 9-year-old girl (P2) was referred to a geneticist due to suspicion of MED. The child is from a twin pregnancy and was delivered via cesarean section at 38 weeks of gestation. Birth weight was 3380 g (0.65 SD) and length was 50 cm (0.68 SD). Following the onset of independent walking at 16 months, progressive varus deformity of the lower limbs was observed, requiring exclusion of a rickets-like disorder. Knee radiographs at 2 years revealed pointed lateral femoral condyles and ossification defects in the medial tibiae, suggesting the presence of Blount disease [12]. At the age of 3, she underwent bilateral proximal corrective tibial osteotomy. At age 5, recurrent gait abnormalities and knee pain with progressive lower limb deformity required right distal femoral hemiepiphysiodesis. By the age of 9, radiographic findings showed avascular necrosis of the femoral heads and changes similar to Legg–Calvé–Perthes disease, raising suspicion of MED (Figure 3). A detailed description and X-ray imaging of the hands, spine, hips, and lower limbs are demonstrated in Supplementary Figure S3.

At the age of 9, her height was 133 cm (0.07 SD) and her weight was 43 kg (1.81 SD). Clinical examination revealed moderate genu valgum, more pronounced on the right, along with lateral instability in the right knee. Additionally, a waddling gait with widely spaced feet was observed. Other findings included internal rotation and varus deviation of the right foot, asymmetry of the scapulae and pelvis, lumbar hyperlordosis, restricted hip abduction, muscle hypotonia, and joint hypermobility (Figure 3).

The novel heterozygous variant c.477+5G>C in *RPL13* identified in the proband through whole-exome sequencing was subsequently confirmed by Sanger sequencing in her brother (a dizygotic twin) and father. Her father was asymptomatic and had normal height—194 cm (2.9 SD). However, he did not undergo comprehensive radiological evaluation. Hip radiographs of her brother demonstrated Perthes-like changes in the proximal femoral epiphysis, which are frequently observed in MED. Detailed clinical and radiological findings of P2’s brother are provided in Supplementary Table S1, Figures S4 and S5.

#### 2.2.3. Family 3

**Figure 4:**
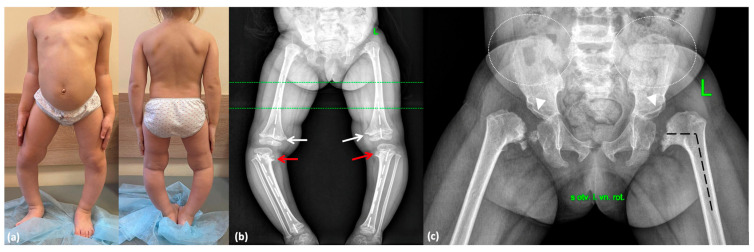
Phenotype and skeletal radiographies of P3 at various ages: (**a**) A photo taken at 3 years 11 months. (**b**) Severe varus deformity (white broken lines) secondary to the Blount-like tibia vara (red arrows) and metaphyseal irregularities predominantly in the medial parts of the femur (white arrows) at 3.5 years. (**c**) Coxa vara (black broken lines), abnormal ossification of the femoral neck, dysplastic acetabulae (white arrowheads), and lace-like appearance of the iliac crests (indicated by white dotted lines) at 3 years and 5 months.

A 4-year-old girl (proband 3; P3) was referred to a geneticist due to suspicion of spondyloepiphyseal dysplasia, which had been observed in her father during his early childhood. The child was born from the second pregnancy as a second-term delivery. Birth weight was 4120 g (1.25 SD), length was 55 cm (2.01 SD), and Apgar score was 8/8. The elder half-sister from the mother’s first marriage, aged 10, is healthy. Hip radiographs showed absence of the femoral head ossific nucleus until the age of 2. Genu varum progressed following the onset of independent walking. Evaluation of biochemical markers of bone metabolism led to the exclusion of a rickets-like disorder. Radiographs at the age of 3.5 years demonstrated SEMD features, including pronounced proximal femoral ossification delay, bilateral femoral neck shortening, varus deformity and ossification defects in the proximal tibial metaphyses, as well as moderate ossification delay in the ventral parts of the vertebral bodies (Figure 4). Temporary hemiepiphysiodesis at the same age was performed on the proximal tibiae.

At the age of 4 years, her height was 90 cm (−2.62 SD) and her weight was 14 kg (−1.43 SD). Additional clinical features included an abnormal gait with internal rotation of the hips and widely spaced feet, genu varum, lumbar hyperlordosis, and hand joint hypermobility (Figure 4).

The previously reported heterozygous variant c.548G>A (p.Arg183His) in *RPL13* was identified through trio whole-genome sequencing. The variant was inherited from her father, who had been under long-term observation for spondyloepiphyseal dysplasia. Clinical and radiological findings of P3’s father are provided in Supplementary Table S1 and Figures S6 and S7.

#### 2.2.4. Family 4

**Figure 5:**
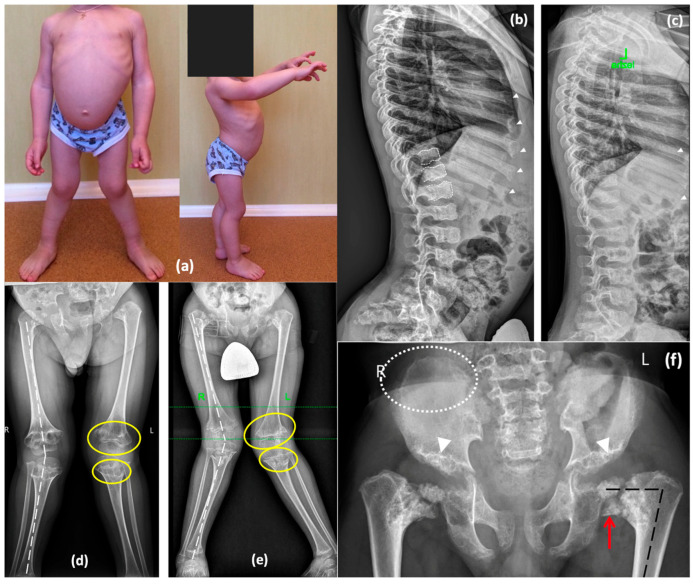
Phenotype and skeletal radiographies of P4 at various ages: (**a**) A photo taken at 3 years. Lateral radiographs of the spine at the age of 5 years (**b**) and 7 years (**c**)—double-convex contour of the endplates (white dotted lines) is moderately represented at the younger age and decreases later; enlarged distal parts of the ribs with rickets-like edges demonstrate the same pattern of improvement (white arrowheads). Lower limb radiographs at the age of 5 years (**d**) and 7 years (**e**) demonstrate progressive valgus deformities (white broken lines) secondary to the remarkable progression of the metaphyseal “chondromatous” changes (indicated by yellow lines). Pelvis radiographs at the age of 7 years (**f**) demonstrate coxa vara (black broken lines) secondary to the shortening and abnormal ossification of the femoral neck and head with Fairbank’s triangle (red arrow), dysplastic acetabulae (white arrowheads), and lace-like appearance of the iliac crests (rounded with the white dotted lines).

A 9-year-old boy (P4), the only child in the family, was referred to a geneticist due to lower limb deformity and short stature. The child was born from the first pregnancy, which was complicated by polyhydramnios. The first delivery was via cesarean section at 37 weeks of gestation due to premature rupture of membranes. Birth weight was 2650 g (−0.8 SD), length was 48 cm (0.29 SD), and the Apgar score was 8/9. Postnatally, shortening of the neck and trunk, a coccygeal passage, and penoscrotal hypospadias were noted, leading to urethroplasty being performed at 1 year and 4 months. A peripheral blood karyotype analysis showed a normal 46,XY male karyotype. Neonatal screening revealed elevated thyroid-stimulating hormone levels, resulting in a diagnosis of congenital hypothyroidism. Therefore, he has been on levothyroxine replacement therapy since he was 3 days old. Hip dysplasia was identified at 4 months. Growth retardation was observed in the first year of life, and spinal deformity progressed by the age of 3 years (Figure 5). Blood lysosomal enzyme levels were tested to exclude Morquio syndrome, and molecular genetic testing was performed to exclude pseudoachondroplasia. Later radiographic findings were consistent with spondyloepimetaphyseal dysplasia, as shown in Figure 5. In addition, specific features such as a lace-like appearance of the iliac crest and central depression of the vertebral endplates (double-humped vertebrae) suggested Smith-McCort dysplasia [13]. At the age of 7, the patient underwent proximal femoral and tibial osteotomies along with bilateral temporary hemiepiphysiodesis of the distal femur.

On examination at the age of 9, significant short stature was noted. His height was 110 cm (−3.75 SD), and his weight was 23 kg (−1.63 SD). Phenotypic features included shortening of the trunk, thoracolumbar S-shaped scoliosis, lumbar hyperlordosis, flexion contractures of the hip joints, genu valgum, and waddling gate. Radiographic findings revealed a progressive worsening of skeletal deformities, especially in the hip joints, while the severity of abnormal ossification in the vertebrae and ribs decreased with age (Figure 5).

As a result, a novel de novo nucleotide variant located in exon 6 of *RPL13*—c.539_541del p.(Ala180_Ser181delinsGly)—was identified in P4 through trio whole-genome sequencing.

## 3. Discussion

SEMD is a heterogeneous group of skeletal disorders characterized predominantly by short stature due to a shortened trunk and lower limb deformities, resulting from combined abnormalities in the vertebrae, epiphyses, and metaphyses. In the latest nosology of genetic skeletal disorders, 32 nosological forms of SEMD with different inheritance patterns have been identified [14]. Despite the similarity in clinical manifestations of SEMD, the underlying molecular causes and developmental mechanisms differ significantly. RPL13-SEMD, inherited in an autosomal dominant manner, is one of the rarest types within this group of disorders. To date, clinical data on 25 patients with RPL13-SEMD from 15 families have been summarized in the literature [5]. It has been shown that postnatal growth retardation was observed in 87.5% of pediatric patients, with 78.6% having a height below −3 SD. Vertebral ossification defects were noted in 81% of patients, lumbar hyperlordosis in 50%, and varus deformity of the lower limbs in 92% of cases. Radiographs of all patients showed abnormal ossification of the epiphyses and metaphyses in the long tubular bones, more prominent in the proximal femurs, with coxa vara formation observed in 94% of cases [5]. Our first three SEMD cases (P1–P3) manifested at 2 years with prominent genu varum and a waddling gait, without significant growth delay, distinguishing them from most reported cases of RPL13-SEMD characterized by severe short stature. Additionally, an absence of femoral head ossification was observed in them during the first year of life. Knee radiographs revealed another hallmark of this SEMD, characteristic of early childhood: a beak-like deformity predominantly affecting the medial parts of the femoral and tibial metaphyses. Consequently, these patients were followed for a period due to suspicion of Blount’s disease. To exclude a rickets-like disorder biochemical markers of bone metabolism was evaluated. At the age of 3, all of these patients underwent surgical correction of knee joint deformities. However, significant ossification abnormalities of the hips, including changes resembling Legg–Calvé–Perthes disease, led to a revision of the diagnosis toward a rare type of SEMD. Analysis of their lateral spine and chest X-rays revealed characteristic ossification patterns of the vertebral bodies, including a mild double contouring of the vertebral endplates, rickets-like widening of the distal rib ends, and a mild lace-like appearance of the iliac crests. These findings characterize milder disease presentations in our patients. A review of the clinical and radiological characteristics in all families with RPL13-SEMD reported to date revealed several cases where growth was either normal or mildly decreased (Table 1).

Notably, a 9-year-old boy, with clinical features similar to P1 and P2, was initially diagnosed with MED due to bilateral hip abnormalities resembling Legg–Calvé–Perthes disease. Subsequently, a heterozygous variant c.477+1G>A in *RPL13* was identified, confirming diagnosis in this patient [15]. A 9-year-old boy with heterozygous variant c.548G>A p.(Arg183His) in *RPL13,* reported by Jacob P. et al. in 2023, had normal height (−0.6 SD) and no genu varum (Table 1) [4]. In contrast, affected members of another family also carrying this variant had significant short stature, ranging from −3.5 SD to −5.8 SD, except for one clinically healthy adult with a height of −2 SD. A similar variant in *RPL13* was found in P3 from the present study, who had moderate growth delay (−2.62 SD) and marked genu varum at the age of 4. Overall, these data confirm the extensive variability in clinical presentations of RPL13-SEMD ranging from asymptomatic to mild and severe forms. A positive family history in P1 and P3, characterized by severe osteoarthritis of the lower limbs and early hips arthroplasty in their parents, played a crucial role in diagnosing autosomal dominant SEMD. It is essential to consider the possible incomplete penetrance of the *RPL13* gene, as demonstrated in Family 2, where a novel c.477+5G>C splice variant was also identified in the proband’s clinically unaffected father. However, without radiological data, his phenotypic status remains uncertain. A similar finding was reported in another family, where the splice variant c.477+1G>T was identified in an unaffected mother and her affected child with a severe form of RPL13-SEMD [3]. This highlights the importance of conducting molecular genetic testing for parents and other family members when planning a pregnancy. To date, 13 nucleotide variants in the *RPL13* gene have been identified, with 6 located in intron 5 and affecting splicing, and 7 missense variants located in exon 6. The localization of these variants represents precise mutational clustering in a highly specific RNA-binding motif, leading to the development of RPL13-SEMD. It is suggested that changes in the nucleotide sequence within this region of *RPL13* may destabilize the C-terminal α-helix of the eL13 protein, leading to ribosome dysfunction [2,3]. Until now, five nucleotide variants altering the donor splice site of intron 5 have been reported, identified in a total of 10 families [2,3,4,5,15]. This study revealed a sixth donor splice site variant, c.477+5G>C, identified in P2 and her family members. Previously, it was proven that the three variants c.477+1G>T, c.477+2T>C, and c.477+3A>T activate the same cryptic splice site, leading to the insertion of 18 amino acids p.(Asn159_Val160ins18) [2,5]. Our splicing analysis of the novel variant c.477+5G>C confirmed a similar alteration in protein structure, suggesting that all variants located in this region may share a common mechanism of splicing disruption. A novel variant c.539_541del p.(Ala180_Ser181delinsGly) located in exon 6 of *RPL13* was identified in P4, consistent with the location of all previously reported missense variants. His clinical features included pronounced disproportionate short stature and genu valgum observed in only three previously reported patients, while genu varum was present in the majority of pediatric patients with RPL13-SEMD [3,4]. His radiographs revealed central depression of the vertebral endplates and an irregular lace-like appearance of the iliac crests, similar to Smith-McCort dysplasia. These findings have been reported in more than half of the RPL13-SEMD cases [5]. Additional features observed in P4, such as genitourinary malformations, including penoscrotal hypospadias, and congenital hypothyroidism, could either be a coincidental association or indicate the clinical variability of RPL13-SEMD, considering the widespread expression of the *RPL13* gene, including the thyroid gland and gonads. However, none of the previously reported patients exhibited extraskeletal manifestations, as the primary focus was on hematological and immune abnormalities common in other ribosomopathies.

## 4. Materials and Methods

### 4.1. Subjects

Four probands and their relatives underwent a comprehensive clinical and radiological evaluation of phenotypic features. Whole-exome sequencing in P2 and trio whole-genome sequencing in families 1, 3, and 4 were performed.

### 4.2. Massive Parallel Sequencing and Variant Analysis

For molecular genetic studies, blood samples were collected from the affected individuals and healthy family members. Genomic DNA was extracted using standard methods. Whole-exome sequencing was performed for P2. For sample preparation, a selective capture method targeting the coding regions of 19,396 genes was used Illumina TruSeq^®^ Exome Kit and IDT xGen^®^ Exome Research Panel (Illumina, San Diego, CA, USA). The average coverage of the whole exome in patients was over ×60; the percentage of target regions with coverage ≥×10 was 99%; and the uniformity of coverage (uniformity Pct > 0.2*mean) was 99%. Primary processing of the sequencing data was performed using the standard automated algorithm provided by Illumina for data analysis, available at https://basespace.illumina.com (accessed on 9 July 2025). Trio whole-genome sequencing was performed for families 1, 3, and 4 on the DNBSEQ-T7 genetic analyzer (MGI Tech Co., Ltd., Shenzhen, China) using paired-end sequencing with a read length of 150 bp (PE150). For sample preparation, a PCR-free method with enzymatic fragmentation (MGI) was used, following the manufacturer’s protocol provided by BGI. Variant annotation was performed using the nomenclature available at http://varnomen.hgvs.org/recommendations/DNA, version 2.15.11. The sequencing data were analyzed using the «NGS-data-Genome» software (https://ngs-data-ccu.epigenetic.ru/main/, accessed on 9 July 2025), created by the Bioinformatics Department of the Research Centre for Medical Genetics, Russia (registration number № 2021662119) [16,17]. Population frequencies and clinical relevance of identified variants were assessed using data from the 1000 Genomes Project, ESP6500, and Genome Aggregation Database (gnomAD) v2.1.1. The clinical significance of the variants was evaluated using ACMG criteria for variant interpretation [11]. To validate the identified nucleotide variants in the affected patient and their family members, Sanger sequencing was performed using the ABI Prism 3500xl Genetic Analyzer (Thermo Fisher Scientific, Waltham, MA, USA). Primer sequences were designed based on the reference sequence of the *RPL13* gene’s target regions (NM_000977.4).

### 4.3. RNA Analysis

Splicing assays were performed for the novel variant NM_000977.3:c.477+5G>C. Total RNA was extracted from peripheral blood mononuclear cells (PBMCs) obtained from P2 and his relatives. PBMCs were isolated using Ficoll gradient centrifugation, and RNA was extracted using the Extract RNA reagent (Evrogen, Moscow, Russia). Reverse transcription was performed using the Reverse Transcription System (Dialat, Moscow, Russia) following the manufacturer’s protocol. The quality of the synthesized cDNA was evaluated by quantitative PCR (qPCR) of the housekeeping B2M gene. To assess the effect of the c.477+5G>C variant on mRNA, the target region of *RPL13* was amplified using the primers 5′-ACGGTTCGGTACCACACGA-3′ and 5′-TGCCGACTGATTCCAAGTCC-3′, followed by Sanger sequencing.

### 4.4. Structural Mapping and Analyses

Structural data for human eL13 (UniProt ID: P26373) were obtained from the Protein Data Bank (PDB: https://www.rcsb.org/structure/6olg, accessed on 9 July 2025). All structures are associated with ribosomal subunits and their assembly into ribosomes and disomes. The representative entry 6OLG (Human ribosome nascent chain complex stalled by a drug-like small molecule), with a resolution of 3.4 Å, was selected for further mutational mapping and analysis.

## 5. Conclusions

Here, we report clinical and radiological characteristics of seven individuals from four unrelated families with rare RPL13-SEMD, associated with two novel and two previously reported nucleotide variants in *RPL13*. Splicing assay was performed for the novel c.477+5G>C variant. The case series presented here has expanded the spectrum of clinical and radiological manifestations in mild forms of RPL13-SEMD, characterized by Blount-like tibia deformity and mild or absent short stature. In P4, severe RPL13-SEMD caused by the novel variant c.539_541del was accompanied by previously unreported extraskeletal manifestations. Therefore, these findings contribute to our understanding of the molecular causes and phenotypic diversity of this novel SEMD type and facilitate the identification of patients with undiagnosed RPL13-SEMD.

## Figures and Tables

**Table 1:** Summary of the clinical and radiological features observed in pediatric patients with RPL13-SEMD and normal stature, based on present and previously reported cases.

Criteria	P1 (Present Study)	P2 (Present Study)	Reinsch B. et al., 2020 [15]	Díaz-González F. et al., 2023 [5]	Jacob P. et al., 2023 [4]
*RPL13* variant	c.477+1G>C	c.477+5G>C	c.477+1G>A	c.477+3A>T	c.548G>A
Mode of inheritance	inherited	inherited	de novo	de novo	de novo
Age (y.o.)	7	9	9	5.7	9
Sex	f	f	m	m	m
Birth length, cm (SDS) ^a^	50 (0.06)	50 (0.68)	51 (0.6)	n/d	n/d
Height at time of examination, cm (SDS) ^a^	115 (−1.31)	133 (0.07)	129 (−0.59)	107 (−1.80)	129 (−0.6)
Referring diagnosis	Rickets-like disease, Blount disease, MED	Rickets-like disease, Blount disease, LCP disease, MED	LCP disease, MED	n/d	n/d
Irregular vertebral bodies	+	+	+	+	+
Platyspondyly	+/−	+/−	−	+/−	+/−
Scoliosis	−	−	−	−	−
Lumbar hyperlordosis	+	+	−	+	−
Short trunk	−	−	−	n/d	−
Metaphyseal involvement	+	+	+	+	+
Delayed epiphyseal ossification	+	+	n/d	+	+
Proximal femoral epiphyseal involvement	+	+	+	+	+
Irregular iliac crests	+/−	+/−	n/d	−	n/d
Coxa vara	+	+	+	+	+
Genu varum	+	+	−	+/−	−
Hyperlaxity	+	+	−	n/d	−
Others	Equino-abducto-plano-valgus foot deformity, cervicothoracic kyphosis	Varus deviation of the right foot	−	Pectus carinatum (mild)	Mild pectus excavatum, thoracic kyphosis
Extraskeletal findings	−	−	−	−	−
Orthopedic surgery	Hemiepiphysiodesis	Hemiepiphysiodesis	−	No but planned for future	n/d

^a^ Patient height SDS was calculated using WHO growth references (http://www.who.int/childgrowth/standards/en/, accessed on 9 July 2025). (+) feature present; (−) feature absent; (+/−) refers to mild changes; n/d—no data; m—male; f—female; LCP disease—Legg–Calvé–Perthes disease.

## Data Availability

Data are contained within the article.

## Supplementary Materials

The supporting information can be downloaded at: https://www.mdpi.com/article/10.3390/ijms26146982/s1.
