# Supplementary material for: Beyond the Known: Expanding the Clinical and Genetic Spectrum of Rare RPL13-Related Spondyloepimetaphyseal Dysplasia
Source: Int J Mol Sci. 2025 Jul 20;26(14):6982. doi: 10.3390/ijms26146982 (PMC12294852; doi:10.3390/ijms26146982)
Supplement: Supplementary file 1 [file ijms-26-06982-s001.zip › ijms-3709198-supplementary.pdf]

Supplementary Table

Table S1. Detailed clinical manifestations in affected relatives of P1-P3 probands

**Mother of P1**	*RPL13* NM_000977.4:c.477+1G>C	P1's mother was examined at the age of 38. She presented with knee and ankle joint pain, walking fatigue, and aching lumbosacral spine pain. From the age of 6-7 years, feet deformities and a waddling gait were noted. At 13 years growth retardation was observed. At 17 years spondyloepiphyseal dysplasia was suspected. At the age of 30, radiography revealed 3rd degree bilateral dysplastic coxarthrosis, gonarthrosis and arthrosis of the ankle joints, genu varum, and widespread spondyloarthrosis of the thoracolumbar spine. Therefore, hips joint replacement was recommended. Upon examination, her height was 143 cm (-3.17 SD), and her body weight was 85 kg (0.95 SD). A Trendelenburg gait was observed, along with persistent adduction contracture of both hip joints, kyphosis in the thoracic spine, lumbar hyperlordosis, varus deformity of the lower limbs, and flat-varus feet.
**Brother of P2 (a dizygotic twin)**	*RPL13* NM_000977.4: c.477+5G>C	P2's brother was examined at the age of 9. His clinical features were characterized by mild pectus carinatum and a waddling gait. His height was 140 cm (+1.41 SD). There were no lower limbs deformities or joint arthralgia. Hip radiography, performed at 9 years after his sister's diagnosis revealed Perthes-like femoral head changes, which is often observed in multiple epiphyseal dysplasia.
**Father of P3**	*RPL13* NM_000977.4: c.548G>A p.(Arg183His)	P3's father was examined at the age of 40 with complaints of back and hip pain. At the age of 7, knee joint pain developed. However, valgus or varus deformity of the lower legs did not occur. At the age of 37, unilateral hip replacement was performed due to severe coxarthrosis. Replacement of the second hip joint is planned. At the time of the examination, the patient's height was 160 cm (-1.34 SD), and lumbar hyperlordosis was observed.

Supplementary Figures

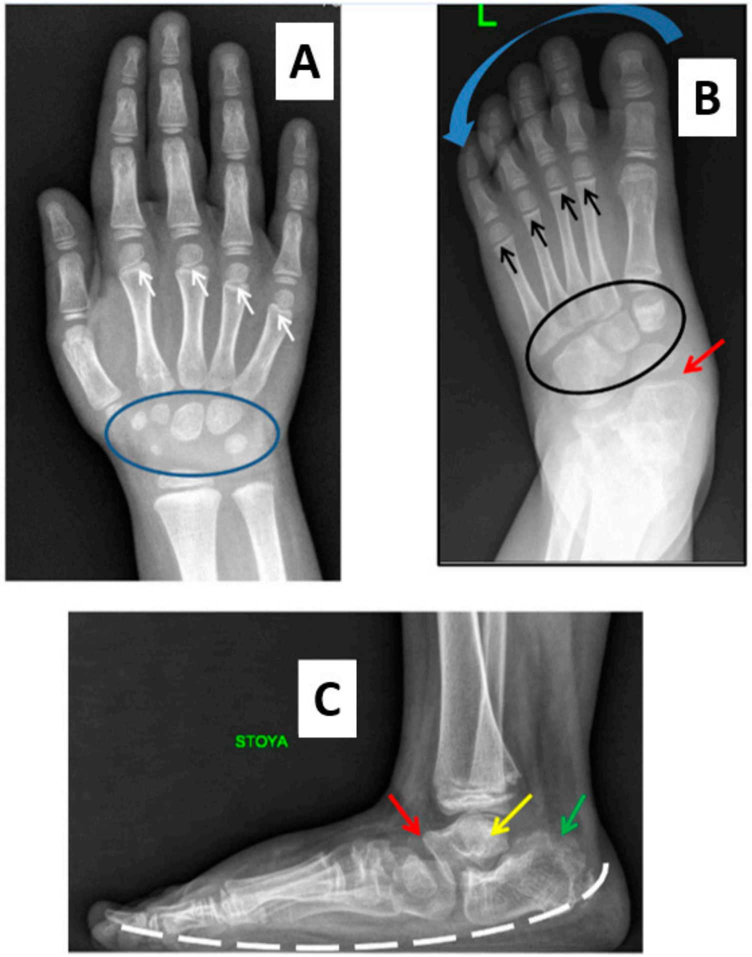

Figure S1. Hand and foot radiographies of P1.

(A) Anteroposterior view of the hand: irregular ossification of the carpal bones with uneven contours (circled with the blue line); irregular margins and cupping of the distal metaphysis of II-V metacarpals (white arrows). (B) Anteroposterior view of the foot: irregular ossification of the midtarsal bones (circled with the black line); irregular margins and cupping of the distal metaphysis of II-V metatarsals (black arrows); flattening of the talar head with lateral navicular subluxation (red arrow) and forefoot abduction (wide blue arrow). (C) Lateral view of the foot flattening and shortening of the talar head with spoor-like dorsal part (red arrow), irregular ossification of the calcaneus (green arrow), deficient ossification of the talar body (yellow arrow); flatfoot deformity (white broken line).

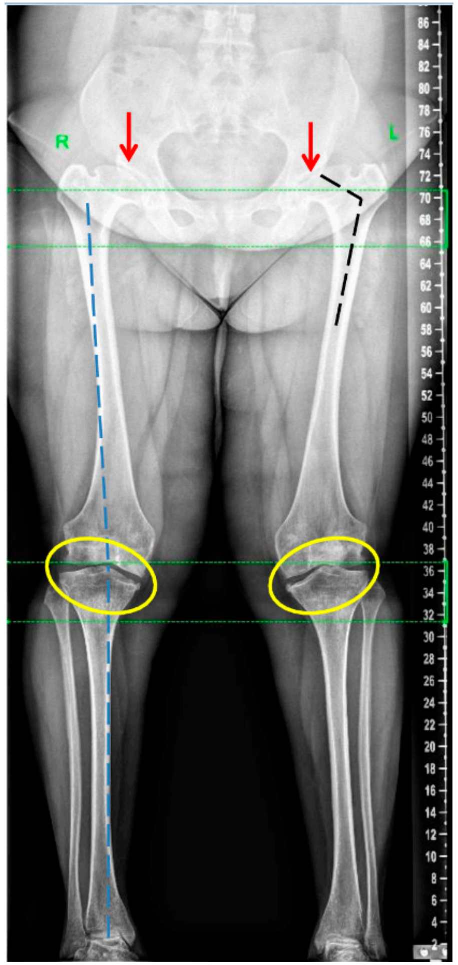

Figure S2. Standing anteroposterior radiography of the lower limbs of P1's mother (38 y.o.)

Mild valgus deformity of the limbs (blue broken line), coxa vara and breva (black broken lines), dysplastic osteoarthritis of the both hips (red arrows), bilateral knee osteoarthritis with valgus joint lines and osteophytes (circled with the yellow line).

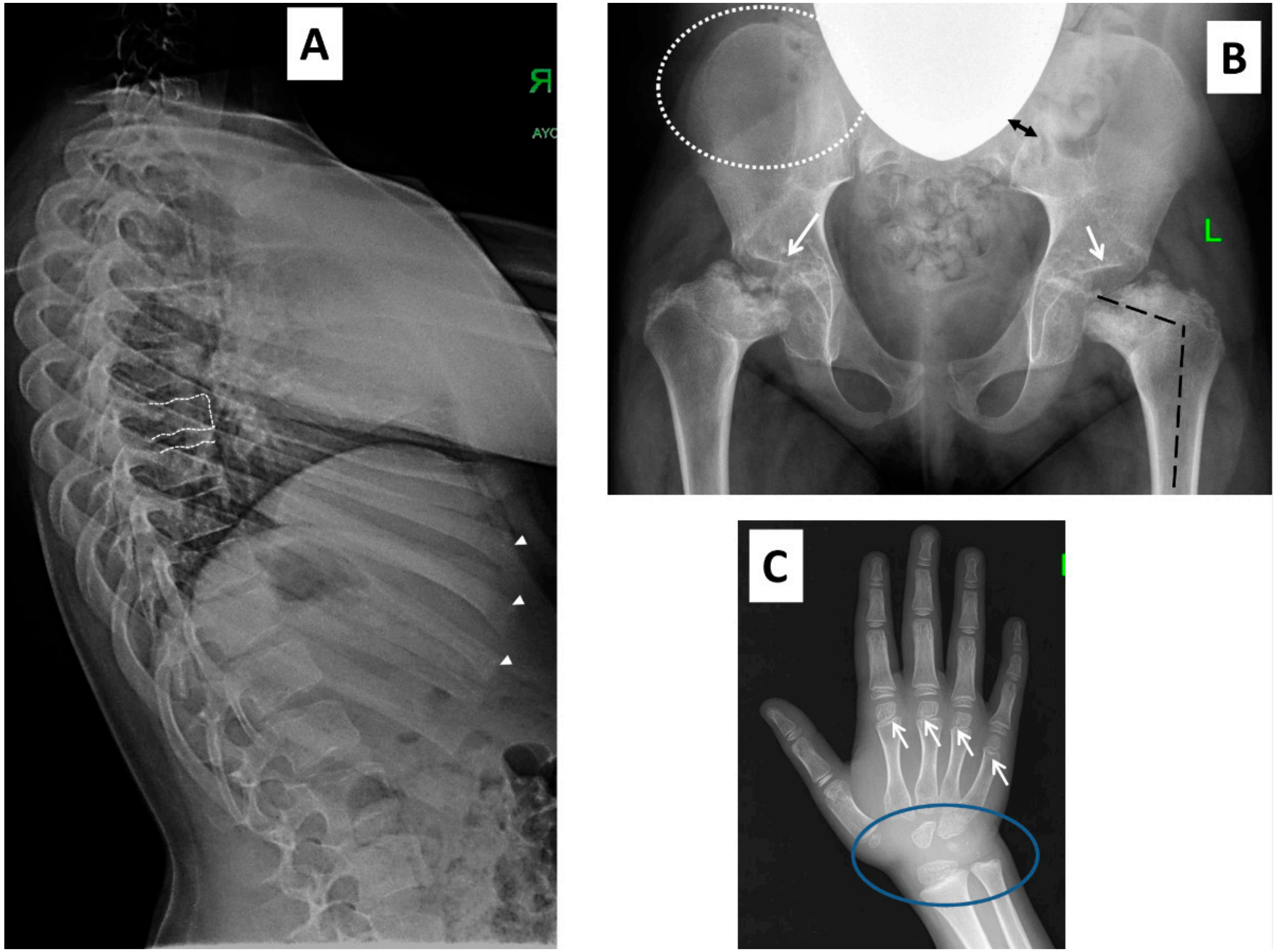

Figure S3. Spine, hips and hand radiographies of P2

A – Lateral radiograph of the spine and the chest: double-convex contour of the cranial and caudal endplates (white dotted lines) is mildly represented, distal parts of the ribs are mildly enlarged (white arrowheads). B – anteroposterior radiography of the hips: coxa vara (black broken lines) secondary to the shortening and abnormal ossification of the femoral neck and head; deficient ossification of pelvis - mildly dysplastic acetabulae (white arrows), widening of the sacroiliac joints (black arrow), minimal laces-like appearance of the iliac crests (rounded with the white dotted lines). C – anteroposterior view of the hand: irregular ossification of the carpal bones with uneven contours (circled with the blue line); irregular margins and cupping of the distal metaphysis of II-V metacarpals (white arrows).

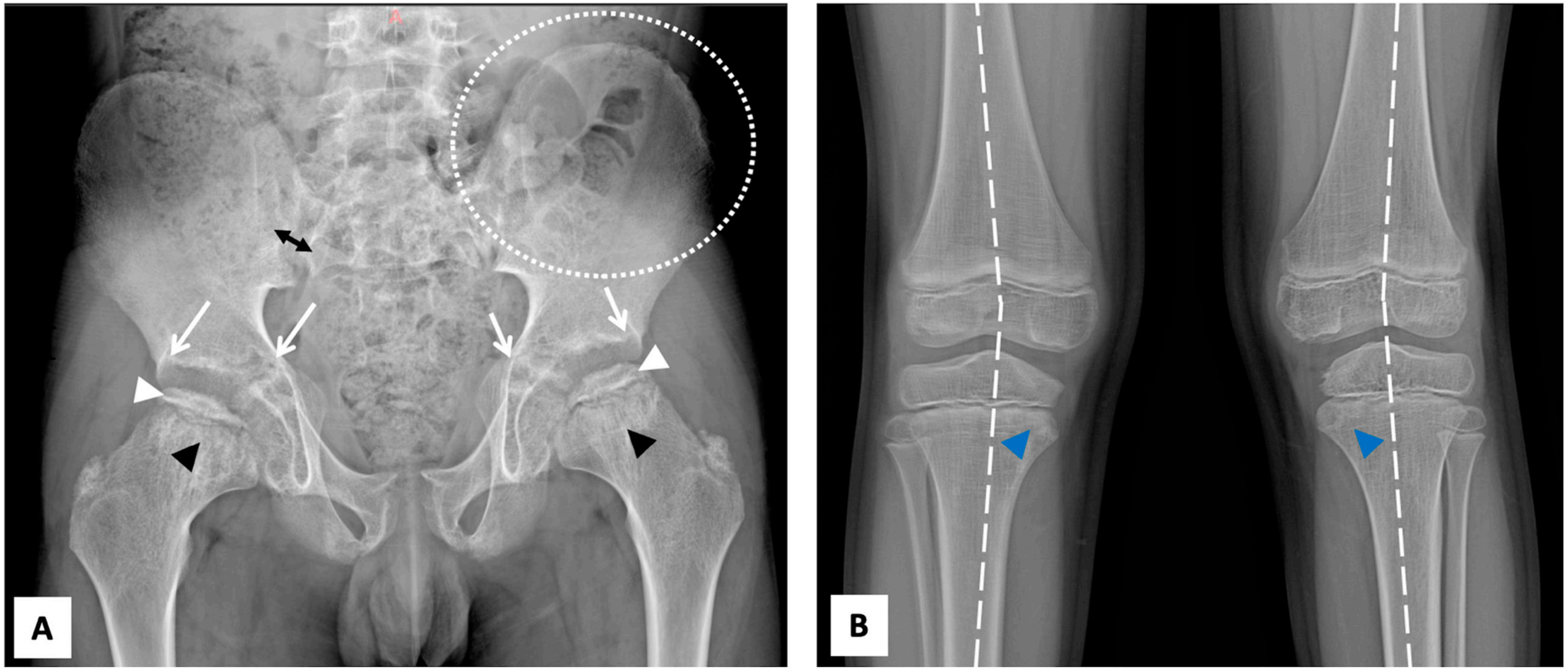

Figure S4. Anteroposterior radiographs of the hips and knee joints of P2’s brother (9 y.o.)

(A) Small flattened femoral epiphyses (white arrowheads), abnormal ossification of the femoral necks (black arrowheads), deficient ossification of pelvis - dysplastic acetabulae (with spur-like margin of underossified triradiate cartilage (white arrow), widening of the sacroiliac joints (black arrow), laces-like appearance of the iliac crests (rounded with the white dotted lines). (B) Mild valgus deformities (white broken lines), metaphyseal “chondromatous” changes (blue arrowheads).

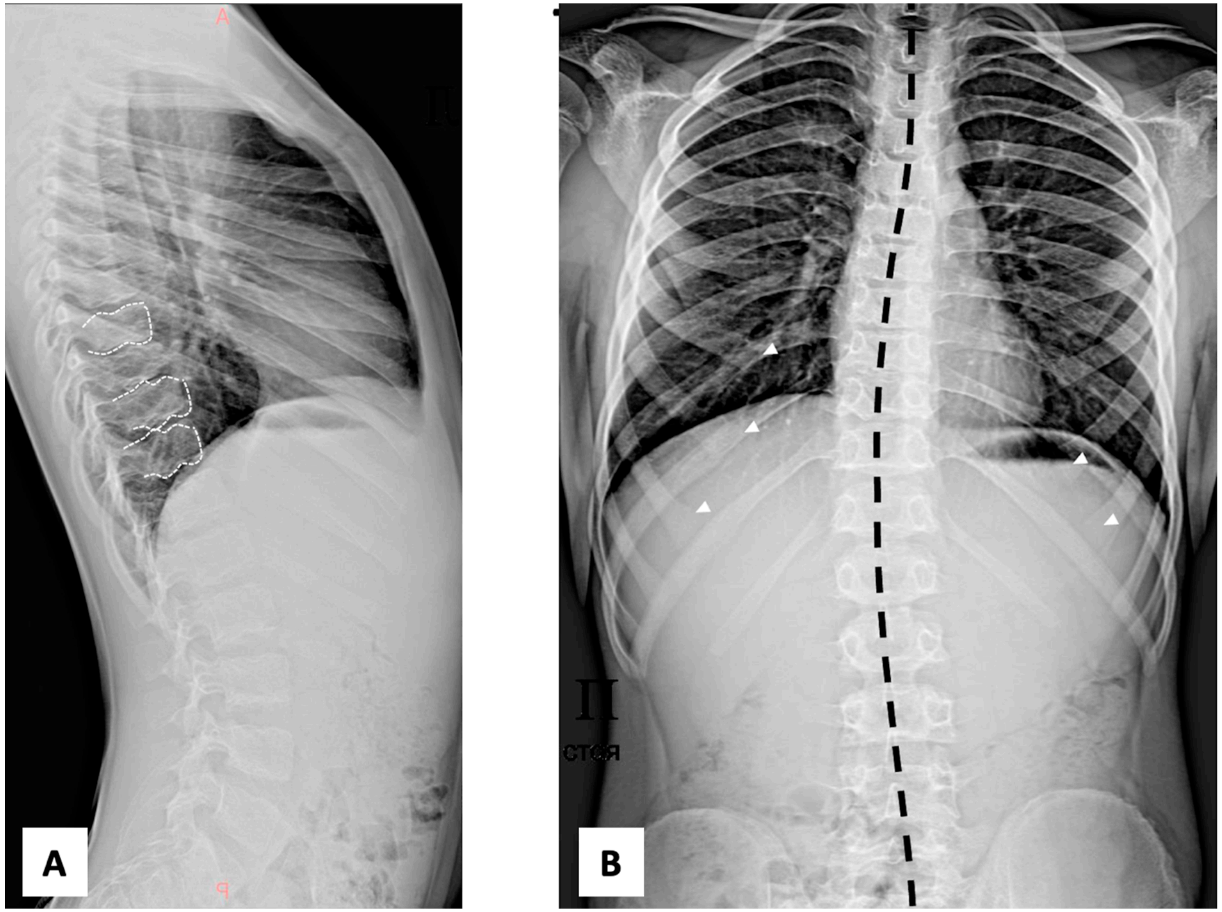

Figure S5. Spinal radiography of P2’s brother

(A) Lateral radiograph of the spine: abnormal ossification pattern of the vertebral bodies - double-convex contour of the cranial and caudal endplates (white dotted lines). (B) Anteroposterior radiograph of the thoracolumbar spine and chest: mild scoliosis (black broken line), enlarged distal parts of the ribs with rickets-like edges (white arrowheads).

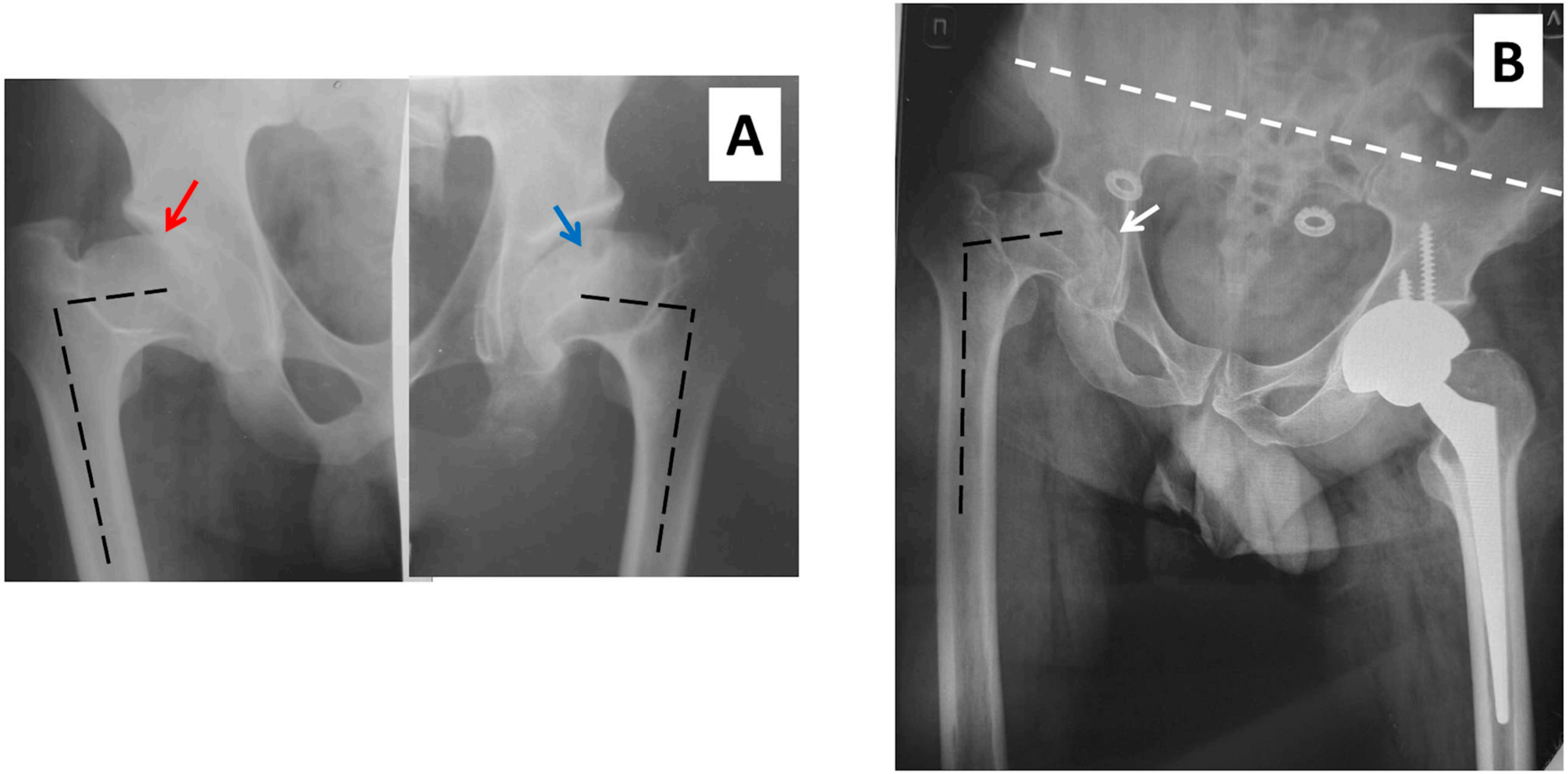

Figure S6. Anteroposterior radiographs of the hips of P3's father

(A) Coxa vara (black broken lines) secondary to the shortening of the femoral neck and mild osteoarthritis of the right hip (narrowing of the articular space, flattened femoral head – red arrow), moderate osteoarthritis of the right hip (more advanced narrowing of the articular space, subchondral cysts – blue arrow). (B) The same patient at the age 37 years - coxa vara (black broken lines), severe osteoarthritis of the right hip (critical narrowing of the articular space, subchondral cysts, osteophytes white arrow), arthroplasty of the left hip, pelvic obliquity secondary to the right hip contracture (white broken line).

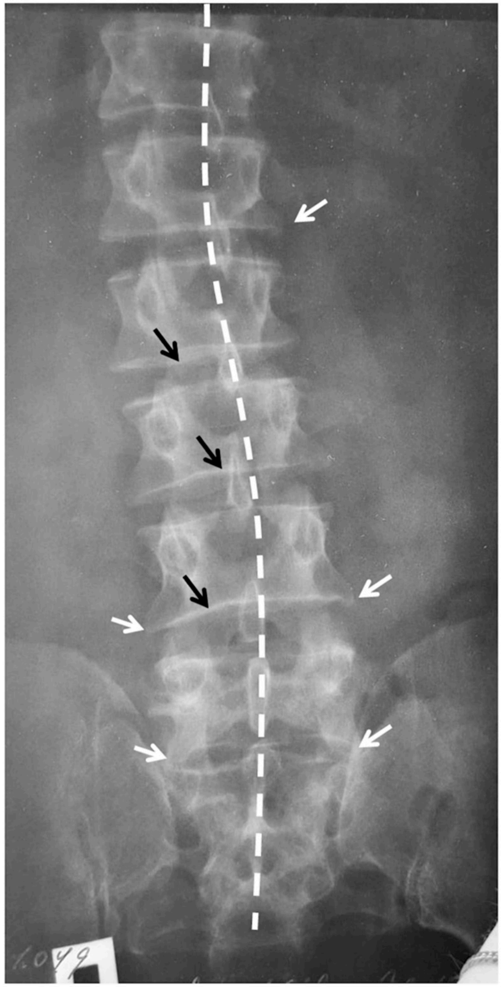

Figure S7. Anteroposterior radiograph of the lumbar spine of P3's father
Radiography demonstrates degenerative changes: osteophytes of the vertebral bodies (white arrows), sclerosis of the endplates (black arrows), mild scoliosis (white broken line).
